# Support for Care Partners and Caregivers: 2023 Summit Session 5

**DOI:** 10.1002/alz.70389

**Published:** 2025-06-19

**Authors:** Kenneth Hepburn, Joseph Gaugler, Karen Roberto, Courtney Van Houtven, Rita Chula, Jason Resendez, Cassandra Thomas

**Affiliations:** ^1^ Nell Hodgson Woodruff School of Nursing Emory University Atlanta USA; ^2^ School of Public Health University of Minnesota Minneapolis Minnesota USA; ^3^ Department of Human Development and Family Science Virginia Tech Blacksburg Virginia USA; ^4^ Department of Population Health Sciences Duke University School of Medicine Durham North Carolina USA; ^5^ American Association of Retired Persons Washington District of Columbia USA; ^6^ National Alliance for Caregiving Washington District of Columbia USA; ^7^ Department of Medicine University of Wisconsin School of Medicine and Public Health at UW Madison Wisconsin USA

**Keywords:** caregiver engagement, dementia care and caregiving, heterogeneity of caregiving, inclusivity, intersectionality

## Abstract

**INTRODUCTION:**

We report on Session 5 of the 2023 NIA National Research Summit on care, services, and supports for persons living with dementia and their care partners/caregivers, “Support for Care Partners and Caregivers.”

**METHODS:**

The session included scholarly presentations, comments by national caregiver advocates, and a conversation among panelists, discussants, and online attendees. Panelists later worked with the National Institute on Aging to identify gaps and opportunities to improve support for care partners and caregivers.

**RESULTS:**

Presentations emphasized the need to construe caregiving within an intersectional framework; include meaningful stakeholder engagement in research; and recognize the complex fabric of local, regional, and national policy affecting caregiving services and supports. Discussants emphasized the need to recognize caregiver needs and enable caregiver agency in research, policy, or practice.

**DISCUSSION:**

The conversation underscored the importance of including caregivers in efforts to improve care/caregiving and by taking as broad a construction of caregiving as possible.

**Highlights:**

Presentations and discussions centered on three key themes:

- The critical importance of integrally engaging family caregivers in all aspects of research, practice delivery and improvement, and policy development;
- Recognition that inclusivity should be a driving principle in identifying and understanding caregivers and the caregiving role;
- Appreciation for the intersection of the spheres of research, practice, and policy in affecting the well‐being of family caregivers and those for whom they provide care.

## 1 INTRODUCTION

Session 5 of the 2023 National Institute on Aging (NIA) Summit explored “caregiving needs, supports, and sources of strength and resilience.” Panelists and discussants were given free range to approach the topic, acknowledging it “can vary by culture, caregiving networks, stage of disease, and living situation.” This report describes the three presentations that formed the main portion of the session, encapsulates the comments of expert discussants, and suggests intersectionality as a conceptual framework that, in retrospect, tied the session together and provides a crosscutting theme for the gaps and opportunities (G&O). The report includes a brief summary of the research G&O that emerged from the session.

## 2 PANEL PRESENTATIONS

The panel presentations were structured to establish a holistic appreciation for the consideration of caregiving needs, supports, and sources of strength. The presentations were framed to establish an appreciation for the heterogeneity of caregiving as a lived experience (Dr. Roberto), illustrate the challenges facing researchers in developing, testing, and scaling effective interventions to support caregiving (Dr. Gaugler), and delineate the larger social and political environment within which caregiving and caregiving research exist (Dr. Van Houtven). The presentations are briefly summarized here.

### 2.1 Informal dementia care: Context matters

This presentation focused on the ways in which informal dementia care occurs within intersecting cultural influences – family structures and norms, informal support, place‐based cultures, and available home‐ and community‐based services – that are themselves affected and shaped by social determinants of health (i.e., education access and quality, health care and quality, neighborhood and built environments, social and community context, and economic stability, as well as demographic characteristics affecting the lived experience, including gender, race, and ethnicity).^1^

The presentation used rurality as an exemplar of family complexity. More persons living with dementia (PLWD) in communities means greater family responsibility. Structures and notions of family are changing, with caregiving boundaries extending beyond the traditional nuclear family. Size of caregiving networks, family expectations about caregiving, and availability and access to services vary by setting (e.g., service acceptance, use, and availability, as well as health system integration with services, are all low in rural settings).^2^, ^3^ Thus, policymakers, health systems, and service organizations need to understand variation across places, recognize complexities within and across dementia caregiving families, and close gaps between care needs and service availability.

### 2.2 Implementation considerations when initiating intervention development

This presentation constituted, through the presentation of the sequenced activities of a specific research project, a call to intervention development researchers to incorporate implementation considerations into the design of their early‐stage activities. Such strategies will inform and enable later National Institutes of Health (NIH) Stage Model^4^ efforts to establish their interventions’ effectiveness through NIH Stage IV and V trials and enable broader implementation of the interventions. The presentation was, in effect, a call to researchers to be mindful, early on, of the challenges of scale‐up and to take full advantage of the opportunities provided by Stage I and II trials. The presentation identified three specific elements of which to be mindful. These include “robust, inclusive, and multi‐level stakeholder engagement” to ensure participation of the intended targets of the intervention in early‐stage work^5^, ^6^, ^7^; the explicit identification and testing of the mechanism of action of the intervention^8^, ^9^; and the use of mixed methods to assess the fidelity, acceptability, feasibility, and appropriateness of the intervention.^10^ Incorporation of these elements, coupled with the use of a formal and explicitly acknowledged adaptation framework (e.g., FRAME^11^), will enable the evolution of an intervention to one whose effectiveness can be tested in later‐stage hybrid effectiveness trials.

### 2.3 Expanding policy supports to promote caregiver resilience and well‐being

This presentation asserted that “Today, caregiving is treated as a private health issue, and caregivers often feel there is no ‘caregiver support system’ to navigate.” This situation derives, in no small part, from the failure of local, state, and national policy to recognize that caregiving is a public health issue^12^, ^13^, ^14^ and takes for granted the enormous social benefits of caregiving (in extended community living for PLWD and reduced healthcare costs and utilization) paid for by the considerable personal toll it exacts from caregivers. Caregivers – primarily women – incur grave economic costs for their efforts: loss of work, wages, and retirement benefits; accumulation of debt; early retirement or withdrawal from the workforce.^15^, ^16^, ^17^ Caregivers of PLWD are among those most affected by these costs because theirs are among the longest and most intensive caregiving roles.^18^

Furthermore, we are unclear about the benefits of public policy to caregivers because most policies are at the local and state levels and are underfunded or inaccessible.^19^ Therefore, there is an opportunity to shape and enact policy that considers caregiving a public health priority. Creating a caregiver‐friendly society will require a multisector approach. Such an approach would create and fund national strategies (e.g., federal paid family leave, caregiver benefits, and appropriations to support the aims of the Reforming American Immigration for Strong Employment [RAISE] Act), expand assessments for screenings and registries, and encourage consumer‐directed programs that allow paying caregivers. Optimal policy would target supports and information to those most at risk of economic calamities (e.g., Black families), systematically identify caregivers during patient visits and integrate them into the healthcare team, expand caregiver training, increase supports to enable caregivers to remain working, and conduct research that identifies causal effects and supports the spread of promising practices.^20^, ^21^, ^22^, ^23^, ^24^, ^25^

## 3 DISCUSSANT COMMENTS

A discussant panel, composed of speakers from AARP, the National Alliance for Caregiving, and the University of Wisconsin, commented on the presentations and added observations on the topic from their perspectives. The conversation that ensued among the panelists, discussants, and a national online audience affirmed the points made by the speakers, especially emphasizing the importance of recognizing the heterogeneous and multifaceted nature of caregiving.

This conversation affirmed the panelists’ observations that, although those who provide care to PLWD provide the “essential human infrastructure” for that care, caregivers are “broadly invisible,” both in policy and in healthcare settings. Subsequent conversations among the authors identified four related points that emerged from this conversation.

### 3.1 Words matter

The term “caregiver” should not be coupled with the term “informal,” a word that, in its suggestion of a casual involvement, fails to appreciate the role's physical, emotional, and financial complexity. There is a need to create a “value vocabulary” around the term “caregiver,” a vocabulary that both recognizes the complexity of the role and the many pathways by which individuals enter into it – by virtue of cultural and/or family values and expectations, a sense of love and connection, or circumstance (the only or nearest option).

### 3.2 Family is no one thing

The panel underscored the need to construe “family” in the broadest possible terms. This is important from the perspective of policy (who might be eligible for benefits, should they be available) and healthcare (providers and systems need to know with whom to interact – and not, for example, to mistakenly exclude non‐kin from crucial conversations). Care generally occurs within large or small networks in which friends, neighbors, and fictive kin may play vital roles as assumed by traditionally defined family members.

**RESEARCH IN CONTEXT**

1. **Systematic review**: The proceedings of this session provide a context for future research on dementia care and caregiving.
2. **Interpretation**: They recommend beginning‐to‐end meaningful involvement of caregivers in research, particularly in early‐stage intervention development efforts, to ensure broader implementation of programs salient to as broad a range of caregivers as possible.
3. **Future directions**: The G&O identified by the session provide direction for future activities in research, practice, and policy.

### 3.3 Caregiver agency is critical

Providers and healthcare systems need to develop a keener appreciation for caregivers' work and be more attentive to ways in which they can support that work. They should also allow their own practices to be informed by caregivers’ knowledge of and insight into the conditions of PLWD. Likewise, caregivers need to be encouraged and supported in their efforts to inform providers of their work and needs and assert the importance of having a voice in the ongoing work of care planning and provision.

### 3.4 Caregiver engagement is essential

The discussants were of one voice in emphasizing the vital need to integrate caregivers’ voices, insights, and perspectives at every step of research and policy development and implementation. They counseled broad inclusion criteria that embraced and accounted for the great diversity of caregivers and caregiving situations.

## 4 DISCUSSION

Taken as a whole, Session 5 of the summit provided a rich context for the G&O derived from it. Likewise, although the concepts named in the session's title, “Caregiving Needs, Supports, and Sources of Strength and Resilience,” were not specifically named as topics in the presentations or discussion, they provided the animating spirit for the session. Thus, along with the points of the presentations and discussion, the session's conclusions can be summarized in three points that integrate the comments of the speakers:

1. *Start with caregivers*. Foster comprehensive inclusion of caregivers in research, intervention development, and implementation, clinical practice, and policy.
2. *Tap into wellsprings of strength*. The session urged the maintenance of as broad a conception and as rich an appreciation of the heterogeneity of caregiving as possible. Heterogeneity almost certainly places some, perhaps many, caregivers in the path of social determinants. Heterogeneity also provides ways of seeing, for individual caregivers and groups of caregivers, cultural, familial, and communal sources that can be tapped to enhance caregiving support and strengthen caregivers’ strength and resilience.
3. *Make healthcare inclusive*. Target caregiver agency and normalization of caregivers as co‐producers of care. Social determinants of health, social outcomes, and a variety of structurally embedded delimiting factors intersect to shape and affect any given caregiving situation, perhaps particularly in the healthcare setting. The advent of new, collaborative dementia care models currently offered by close to 400 healthcare providers that must identify, include, and assist care partners of patients with dementia (i.e., the Guiding an Improved Dementia Care Experience/GUIDE)^26^ may offer opportunities to expand dementia care identification across healthcare systems.

Intersectionality asserts that individuals’ experiences derive from the overlapping influences of individual social position (e.g., race, class, gender) and larger social, economic, and political systems (e.g., racism).^27^ The various points made by the panelists and during the discussion and conversation that followed underscored how interrelated caregiving issues were and how essential it was to bring a perspective of intersectionality to bear on every facet of the topic. The overall conversation consistently recognized that every aspect of caregiving required and benefited from a nuanced appreciation for the multiple interacting and often conflicting forces that shape and affect it.

The points that emerged from the session provided grist for further conversations, and this session offered an important platform for raising and addressing them. Researchers, for example, might embrace the seemingly simple notion of “start with caregivers.” Attempts to operationalize it, however, will expose issues that will require difficult conversations. Who defines a caregiver? If a class of persons is not specifically named in policy or healthcare coverage, how should they be included? What are the parameters of diversity? How inclusive of class, race, gender, ethnicity, geography, and so forth can and must research be to represent heterogeneity? What methods and designs can enable the integration of results across studies? How can results from studies of urban, minoritized caregivers, affluent, dominant‐race caregivers, and rural, low‐income immigrant caregivers (for example) be designed to enable meaningful cross‐fertilization and interpretation?

### 4.1 G&O

The full statements of the research G&O identified by this and the other seven summit sessions can be found in the Summit Report (https://www.nia.nih.gov/2023‐dementia‐care‐summit#gaps). These G&O were developed following the summit through a set of conversations between the authors and the summit organizers. Below, we briefly describe those identified in Session 5 and the rationale for their inclusion among the G&O:

1. Although it is relatively easy to identify PLWD from electronic health records, the capacity to identify their family caregivers from these records is very limited. Therefore, G&O 5.1 suggests that to provide caregivers with access to support services and research opportunities, health systems should find or improve ways to identify caregivers of PLWD who are patients of the system and to identify patients who identify themselves as playing caregiving roles.
2. Panelists and discussants underscored the heterogeneity of caregiving situations. Therefore, G&O 5.2 emphasizes the need to design and conduct culturally informed research on all aspects of caregiving (e.g., “caregivers’ physical, emotional, and financial well‐being and the provision of caregiving supports and services”).
3. Finally, the panel acknowledged that the provision of supports and services occurs against a backdrop of “systems‐level policies and practices at the local, state, and national levels” that affect the physical, emotional, financial, and well‐being of PLWD and their caregivers. Therefore, G&O 5.3 directs us to take a broad and inclusive research and policy analytic view across the system and multiple levels of government/policy structures to examine their effects – very likely differential – on PLWD/caregiver capacity and well‐being.

## CONFLICT OF INTEREST STATEMENT

The authors declare no conflicts of interest. Author disclosures are available in the Supporting Information.

## CONSENT STATEMENT

Consent was not necessary.

## Supporting information



Supporting Information
